# Programmable Self-Assembly of RNA Nanostructures with >100 Unique Components

**DOI:** 10.64898/2026.09.02.748990

**Published:** 2026-09-03

**Authors:** Liangxiao Chen, Zhishang Li, Jun Yan, Swarup Dey, Cong Li, Alexandra Petrova, Abhay Prasad, Deeksha Satyabola, Kira DeVore, Xinyi Tu, Yanzhe Qu, Shiyumou Wang, Gengshi Wu, Po-Lin Chiu, Di Wen, Joseph Che-Yen Wang, Hao Yan, Peng Yin, Di Liu

## Abstract

Sophisticated biomolecular functions often arise from large, precisely organized architectures, motivating efforts to construct increasingly complex structures through programmed nucleic acid self-assembly. Although RNA offers a richer repertoire of structural motifs and biological functions than DNA, RNA nanostructures constructed to date have remained substantially less complex and less scalable than their DNA counterparts, most notably DNA origami and DNA bricks, which rely on large libraries of synthetic single-stranded (ss) DNA strands. Directly adapting these strategies to RNA, however, faces two major barriers: (i) the high cost of producing large libraries of distinct synthetic ssRNA strands, and (ii) the limited stability of ssRNA under conditions commonly used for assembling multicomponent nucleic acid nanostructures. Here we present the double-stranded RNA (dsRNA) bricks approach, which addresses both barriers by adopting principles inspired by natural RNA systems: (i) many distinct RNA components (“bricks”) are encoded within a single precursor transcript and released by enzymatic processing, (ii) each brick’s predominantly double-stranded nature enhances stability. The resulting dsRNA bricks self-assemble through programmable branched kissing-loop interactions. Using this approach, we constructed complex two- and three-dimensional RNA nanostructures with more than 100 distinct components, representing, to our knowledge, the largest and most compositionally complex RNA nanoarchitectures reported to date. Overall, the dsRNA bricks approach establishes a scalable and robust framework for constructing complex RNA nanostructures with compositional and architectural sophistication approaching that of DNA-based systems, thereby opening new opportunities for programmable RNA materials and RNA-based devices.

## Full Text Availability

The license terms selected by the author(s) for this preprint version do not permit archiving in PMC. The full text is available from the preprint server.

